# Relationship between household wealth inequality and chronic childhood under-nutrition in Bangladesh

**DOI:** 10.1186/1475-9276-5-15

**Published:** 2006-12-05

**Authors:** Rathavuth Hong, James E Banta, Jose A Betancourt

**Affiliations:** 1 George Washington University, School of Public Health and Health Services, 2300 Eye Street N.W. Washington, DC 20037, USA; 2 Academy of Health Sciences, U.S. Army Medical Department Center & School, 2250 Stanley Road, Fort Sam Houston, TX 78234, USA

## Abstract

**Background:**

Household food insecurity and under-nutrition remain critically important in developing countries struggling to emerge from the scourge of poverty, where historically, improvements in economic conditions have benefited only certain privileged groups, causing growing inequality in health and healthcare among the population.

**Methods:**

Utilizing information from 5,977 children aged 0-59 months included in the 2004 Bangladesh Demographic and Health Survey , this study examined the relationship between household wealth inequality and chronic childhood under-nutrition. A child is defined as being chronically undernourished or whose growth rate is adversely stunted, if his or her z-score of height-for-age is more than two standard deviations below the median of international reference. Household wealth status is measured by an established index based on household ownership of durable assets. This study utilized multivariate logistic regressions to estimate the effect of household wealth status on adverse childhood growth rate.

**Results:**

The results indicate that children in the poorest 20% of households are more than three time as likely to suffer from adverse growth rate stunting as children from the wealthiest 20% of households (OR=3.6; 95% CI: 3.0, 4.3). The effect of household wealth status remain significantly large when the analysis was adjusted for a child's multiple birth status, age, gender, antenatal care, delivery assistance, birth order, and duration that the child was breastfed; mother's age at childbirth, nutritional status, education; household access to safe drinking water, arsenic in drinking water, access to a hygienic toilet facility, cooking fuel cleanliness, residence, and geographic location (OR=2.4; 95% CI: 1.8, 3.2).

**Conclusion:**

This study concludes that household wealth inequality is strongly associated with childhood adverse growth rate stunting. Reducing poverty and making services more available and accessible to the poor are essential to improving overall childhood health and nutritional status in Bangladesh.

## Background

In spite of remarkable advances in public health during recent decades, many people throughout the developing world remain vulnerable to food insecurity, under-nutrition, and ill health [1]. These problems tend to be particularly severe in developing countries struggling to emerge from the scourge of extreme poverty [2]. In such countries, the health and nutritional benefits spawning from economic growth tend to be concentrated only among the economically-advantaged sectors of the population [3-5].

The Bangladesh economy has improved over the recent past. The country's substantial agricultural sector contributes to 19% of the overall gross domestic product (GDP) and to the significant increase of all exported agriculture products. The industrial sector is rapidly becoming one of the more important components of the Bangladesh economy, contributing 34% to GDP, while the service sector contributing to 47% of GDP. However, despite these economic improvements, the country still struggles to emerge from the clutches of poverty. Almost four in every ten people (36%) live below the absolute poverty line with incomes less than $1 per day. Most reside in rural areas and those living in urban areas lack many basic amenities. A significant proportion of the population does not have sufficient access to food, sanitation facilities, or health care [6,7]. According to the 1998 Bangladesh Bureau of Statistics, approximately 2.4 million households (or 24 million people) have an energy intake of less than 1,805 kcal per person per day: an indicator of extreme poverty [8]. Recent improvements in economic conditions are believed to have benefited mainly the wealthier sector of the population more so than the less wealthy sector, with the effect of this widely and seemingly growing economic inequality in health and nutrition still very poorly understood [9]. Bangladesh is similar to many other developing countries: under-nutrition is one of the leading causes of childhood morbidity and mortality. Under-nutrition among children is often caused by the combined effects of improper or insufficient food intake, repeated episodes of infections, and inadequate care during sickness [10]. Additionally, under-nutrition affects somatic growth, impairs the immune system, and increases the risk of infection [11-13]. In developing countries around the world, an estimated 46 million children are malnourished, 127 million are underweight, and 148 million children are adversely growth rate stunted [14]. A recent comparative risk assessment by the World Health Organization estimates under-nutrition is by far the largest contributor to the global burden of disease [15].

Previous research has associated childhood nutrition with a child's multiple-birth status, a mother's education and nutritional status, a father's employment, the mother's breastfeeding and feeding practices, access to safe drinking water and sanitation facilities, access to health care, prevalence of parasitic and infectious diseases, parent's health-seeking behavior, race or ethnicity, rural residence, and social network and family support [16-21]. Demographic characteristics such as a child's age and gender, birth interval (both preceding and succeeding), and the mother's age at childbirth, have also been associated with child nutrition status [22].

According to Kawachi, economic inequality is an independent determinant for childhood under-nutrition [9]. Countries with a greater degree of economic inequality tend to have an overall poorer average population health status than countries with more economic equality [1,23]. Suffice it to say that the relationship between economic inequality and under-nutrition is complex. This is in part due to the fact that greater national wealth does not necessarily translate into better health care for all. If that were the case, then the single best approach to improving health care would be to maximize economic growth [24]. Additionally, economic growth does not always benefit all sections of the society equally. A country's social and economic inequality affects food availability, access to health services, and disease morbidity and mortality among the many sections of a society differently. In Japan, for example, a rapid improvement in life expectancy in the last few decades was associated not only with its rapid economic growth, but also with a low level of economic inequality [25].

A number of studies have illustrated that children from poorer households tend to be more undernourished than children in wealthier households [4,5,26-28]. Social deprivation has also been linked with a child's nutritional status [29]. However, the relationship between economic inequality and a child's nutritional status is not conclusive. A recent study in Mexico discovered that household poverty is not a necessary condition for children to be undernourished [17]. Another recent study in Ecuador found inconsistent evidence to indicate any relationship between economic inequality and the nutritional status of children [9]. Additionally, a study in Cambodia found that acute under-nutrition in children was associated with a mother's feeding practices, parent's health-seeking behavior, and personal hygiene; however, there was no association with household food insecurity [16]. The primary objective of this study is to investigate the association between household wealth inequality and childhood under-nutrition in Bangladesh. We will also examine the effects of other potential risks and confounding factors on childhood under-nutrition.

## Methods

The analysis in this study is based on 5,977 children aged 0–59 months with valid information on length or height included in the 2004 Bangladesh Demographic and Health Survey (BDHS). Children whose information on length/height was missing (867) or invalid (130) were excluded. The BDHS collected demographic, socioeconomic, and health data from a nationally-representative sample of 11,440 women aged 15–49 (98.6% of eligible women) from 10,500 households (99.8% of eligible households) included in the survey. The study contained 3,513 households from urban areas and 6,987 household from rural areas. The sampling design allowed for national estimates and division-level estimates from the six divisions of all demographic and health indicators collected in the survey. The master sampling frame for the BDHS was based on the 2001 national census. The sampling design was a multistage cluster sample consisting of 361 primary sampling units (PSUs): 122 from urban areas and 239 from rural areas. An average of 30 households was selected per PSU. Details of the sampling design are provided in the main BDHS report [30].

To assess the physical growth and nutritional status of children, the survey measured height or length and weight of all children aged 0–59 months. Details about these measurements are included in the main survey report. The ratio of the height and age of a child serves as a good proxy for chronic under-nutrition among children, and it is not significantly affected by a child's recent episodes of illness. Children with a z-score of height-for-age more than 2 standard deviations below the international referenced median established by the World Health Organization are defined as stunted [31,32].

The BDHS also includes a household wealth status index which is estimated from several household characteristics and asset variables using a principle component analysis [33]. The household characteristics used to estimate the wealth index include having electricity, type of source of drinking water, access to a sanitation facility, availability of cooking fuel, main roof material, main wall material, floor material. The asset variables include durable goods (wardrobe, table, chair or bench, watch or clock, radio, television, bicycle, motorcycle, sewing machine, and telephone) and land ownership [30]. This household wealth index is used as a proxy indicator for household wealth status in this analysis. Household wealth inequality is measured by dividing the wealth index into quintiles, with the lowest quintile representing the poorest 20% of households and the highest quintile representing the wealthiest 20% of households in Bangladesh.

The analysis conducted in this study adjusts for the effects of potentially confounding factors due to the fact that household wealth status is correlated with maternal nutrition and other socioeconomic and demographic factors that can also affect the nutritional status of children [34,35]. These potentially confounding factors include children of multiple-births (single-born, twin or higher order), a child's age (0–11, 12–23, 24–35, 36–47, 48–59 months), child's gender (boy, girl), birth order (1, 2, 3, 4+), mother's access to antenatal care (no, yes), availability of professional assistance at delivery (no, yes), duration of breastfeeding (never breastfed, 0–11, 12–17, 18–23, ≥ 24 months); mother's age at childbirth (15–24, 25–34, 35–49 years), mother's body mass index (BMI) (18.5–24.9, < 18.5, ≥ 25.0 kg/m^2^), mother's education (no education, primary or less, secondary or higher); household access to safe drinking water (no, yes), presence of arsenic in drinking water (≤ 50, > 50 parts per billion), access to a toilet facility (no, yes), cleanliness of cooking fuel (not highly polluted, highly polluted) residence (urban, rural), and geographic location (Barisal, Chittagong, Dhaka, Khulna, Rajshahi, Sylhet). For further details on the variable definitions, please see Table 1.

**Table 1:** Sample distribution and prevalence of stunting among children age 0–59 months by household wealth status and other selected characteristics, Bangladesh 2004

**Characteristic**	**Weighted number of children**	**Percent distribution of children**	**Prevalence of stunting**
**Bangladesh**	5,977	--	43.0
**Wealth status**			*(p = 0.000)*
5^th ^quintile (richest)	989	16.6	25.1
4^th ^quintile	1,081	18.1	39.8
3^rd ^quintile	1,176	19.7	42.5
2^nd ^quintile	1,229	20.6	47.0
1^st ^quintile (poorest)	1,502	25.1	54.4
**Child of multiple birth**			*(p = 0.000)*
Single-born	5,903	98.8	42.8
Twin or higher order	74	1.2	62.8
**Child's age (month)**			*(p = 0.000)*
0–11	1,145	19.2	17.3
12–23	1,182	19.8	50.9
24–35	1,205	20.2	44.7
36–47	1,239	20.7	49.3
48–59	1,207	20.2	51.5
**Child's sex**			*(p = 0.231)*
Boy	3,036	50.8	42.6
Girl	2,940	49.2	43.5
**Antenatal care**			*(p = 0.000)*
No	3,629	60.7	49.9
Yes	2,348	39.3	32.4
**Delivery assisted by health professional**			*(p = 0.000)*
No	5,183	86.7	45.5
Yes	793	13.3	26.8
**Child's birth order**			(p = 0.000)
1	1,709	28.6	40.1
2	1,538	25.7	38.8
3	1,114	18.6	42.2
4+	1,615	27.0	50.7
**Breastfeeding status (month)**			*(p = 0.000)*
Never	10	0.2	54.9
0–11	1,372	23.0	21.0
12–17	903	15.1	48.3
18–23	867	14.5	56.0
≥ 24	2,824	47.3	48.0
**Mother's age at childbirth (year)**			*(p = 0.005)*
15–24	3,735	62.5	42.8
25–34	1,894	31.7	42.0
35–49	348	5.8	50.5
**Mother's BMI (kg/m^2^)**			*(p = 0.000)*
< 18.5	2,227	37.3	50.4
18.5–24.9	3,399	56.9	40.2
≥ 25.0	325	5.4	21.1
**Mother's education**			*(p = 0.000)*
No education	2,236	37.4	50.4
Primary or less	1,875	31.4	45.6
Secondary or higher	1,866	31.2	31.6
Safe drinking water*			*(p = 0.769)*
No	377	6.3	42.3
Yes	5,595	93.6	43.1
**Arsenic in drinking water (parts per billion)**			*(p = 0.874)*
≤ 50	5,399	90.3	42.8
> 50	563	9.4	45.1
**Hygienic toilet^†^**			*(p = 0.000)*
No	2,579	43.2	50.0
Yes	3,393	56.8	37.7
**Cooking fuel^‡^**			*(p = 0.000)*
Not highly polluted	402	6.7	29.2
Highly polluted	5,047	84.5	44.8
**Urban/rural**			*(p = 0.000)*
Urban	1,174	19.6	37.7
Rural	4,803	80.4	44.3
**Geographic division**			*(p = 0.000)*
Barisal	355	5.9	49.0
Chittagong	1,325	22.2	46.3
Dhaka	1,834	30.7	44.6
Khulna	649	10.9	32.0
Rajshahi	1,323	22.1	40.2
Sylhet	490	8.2	46.2

* Safe sources of drinking water include piped water and tube well.

^†^Hygienic toilet includes toilet connecting to sewage or having a septic tank and pit latrine.

^‡ ^Highly polluted cooking fuels include straw, wood, and animal dung.

The effects of household wealth status and other factors on a child's growth-stunting were estimated using multivariate logistic regression methods using the analytical software package STATA [36]. We also analyzed alternative regression models separately for boys and girls, and for urban and rural to assess the relative significance of different confounding factors among these groups. In our analysis, we assigned assorted weights to restore the representativeness of the sample, adjusting for non-response bias and over-sampling in certain categories of respondents such as among those respondents living in the rural areas [30]. The results are presented as percent of stunting and significant level (p-value) in bivariate analysis and odds-ratios (OR) with 95% confidence intervals (CI) logistic regression analysis.

## Results

Twenty-five percent of children aged 0–59 months in Bangladesh live in the poorest 20% of households while 17% live in the wealthiest 20% of households (Table 1). Slightly more than one percent of Bangladeshi children were born of multiple-births. Children are almost equally distributed by age and gender. Thirty-nine percent of Bangladeshi children have mothers who received antenatal care during their pregnancy and 13% of them were delivered by a health professional. Twenty-nine percent of children are first-order births while another 27% are fourth-order births or higher. Almost all Bangladeshi children are breastfed with more than three-quarters (77%) being breastfed for one year or longer. The majority (63%) of all children were born to mothers aged 15–24. Fifty-seven percent of children sampled have mothers with a normal body weight while 37% of mothers are underweight and 5% are overweight. Approximately one-third (37%) of mothers have no education with 31% having a primary education and secondary or more education respectively. About nine in every ten children live in households with safe sources of drinking water (piped or tube-well), 9% live in a household where the source of drinking water contains arsenic (> 50 parts per billion), 57% live in a household with access to a toilet facility, and 85% live in households using highly-polluted cooking fuels. About one in every five children lives in urban areas. By geographic division, 31% of Bangladeshi children live in the Dhaka division, 22% in the Chittagon and Rajshahi divisions respectively, and 11% in the Khulna division. Only 6% of the children live in the Barisal division with another 8% living in the Sylhet division.

Overall, 43% of Bangladeshi children aged 0–59 months are adversely growth-rate stunted (Table 1). This figure represents one of the highest rates of chronic childhood under-nutrition in the South Asian region and in fact, in the world [8]. The prevalence of childhood growth-stunting declines as the household wealth status increases, from 54% in the poorest households (lowest wealth index quintile) to 25% in the wealthiest households (highest wealth index quintile). This prevalence of childhood growth-stunting is higher among multiple-birth children and increases with a child's age. The prevalence is considerably less common during the first 12 months of life when most babies are fully breastfed than at older ages. The prevalence rapidly increases from 12–23 months of age, after which it levels off with a slight fluctuation. The prevalence of childhood growth-stunting does not vary much by a child's gender. It is higher however, among children whose mother did not receive antenatal care and delivered without professional assistance. The prevalence is somewhat higher among children of fourth-order births or higher.

Among children who were breastfed, the prevalence of growth-stunting increases if a child is breastfed for longer than 11 months (48%–56%). The prevalence of growth-stunting is also higher among children who were never breastfed (55%). Additionally, children of older mothers are more likely to suffer from growth-stunting than those whose mothers are in a younger age group. Also the prevalence of adverse growth-stunting is strongly negatively associated with a mother's BMI and educational status.

Children from households without access to a toilet facility are more likely to suffer from growth-stunting (50%) than in households with access to a toilet facility (38%). Additionally, children in households where highly-polluted cooking fuels are used are more likely to suffer from growth-stunting than in households where clean cooking fuels are used. However, the results indicate that the availability of safe drinking water and the presence of arsenic in drinking water are not associated with the prevalence of growth-stunting. The prevalence of growth-stunting is lower in urban areas (38%) than in rural areas (44%), and is much lower in Khulna division (32%) than other divisions of Bangladesh (40–49%).

### Effects of wealth status on growth-stunting

The unadjusted odds of suffering from growth-stunting are 3.6 times higher among children living in the poorest (lowest wealth index quintile) households than among children in the wealthiest (highest wealth index quintile) households (OR = 3.6; 95% CI: 3.0, 4.3) (Table 2, Model 1). The odds of suffering from childhood growth-stunting declines consistently as wealth index increases. This relationship remains strong even when controlling for a child's multiple birth-status, age, gender, antenatal care, type of delivery, birth order, and duration of breastfeeding. In Model 2, when these childhood characteristics are controlled for, the odds of a child suffering from growth-stunting are 2.7 times higher among the poorest 20% of households than in the wealthiest 20% of households. Additionally, controlling for a mother's characteristics such as age at childbirth, BMI, and education slightly reduces the effect of wealth status. In the full model (Model 4), when we control for child's and mother's characteristics and the availability of safe drinking water, arsenic in drinking water, access to toilet facility, clean cooking fuel, urban/rural residence, and geographic division, the effect of household wealth status on childhood growth-stunting remains large and highly, statistically significant (OR = 2.4; 95% CI: 1.8, 3.2).

**Table 2:** Effects of household wealth status and other selected characteristics on stunting among children age 0–59 months, Bangladesh 2004

Variable	**OR (95% CI)**			
	
	**Model 1**	**Model 2**	**Model 3**	**Model 4**
**Wealth status**				
5^th ^quintile (richest)^†^	--	--	--	--
4^th ^quintile	2.0 (1.6, 2.4)	1.8 (1.4, 2.2)	1.6 (1.3, 2.0)	1.7 (1.3, 2.2)
3^rd ^quintile	2.2 (1.8, 2.7)	1.9 (1.5, 2.3)	1.6 (1.3, 2.0)	1.8 (1.3, 2.3)
2^nd ^quintile	2.6 (2.2, 3.2)	2.1 (1.7, 2.6)	1.7 (1.4, 2.2)	1.9 (1.4, 2.5)
1^st ^quintile (poorest)	3.6 (3.0, 4.3)	2.7 (2.2, 3.4)	2.1 (1.7, 2.7)	2.4 (1.8, 3.2)
**Child of multiple birth**				
Single-born^†^		--	--	--
Twin or higher order		2.9 (1.6, 5.1)	3.1 (1.8, 5.5)	3.6 (2.1, 6.3)
**Child's age (month)**				
0–11^†^		--	--	--
12–23		4.1 (2.7, 6.1)	4.4 (2.9, 6.5)	5.2 (3.4, 8.1)
24–35		3.1 (2.1, 4.5)	3.4 (2.3, 4.9)	3.7 (2.5, 5.6)
36–47		3.5 (2.4, 5.1)	3.9 (2.7, 5.8)	4.5 (3.0, 6.7)
48–59		3.8 (2.6, 5.5)	4.2 (2.9, 6.1)	4.9 (3.3, 7.3)
**Child's sex**				
Boy^†^		--	--	--
Girl		1.1 (1.0, 1.2)	1.1 (1.0, 1.2)	1.0 (0.9, 1.2)
**Antenatal care**				
No^†^		--	--	--
Yes		0.7 (0.6, 0.8)	0.7 (0.6, 0.9)	0.8 (0.6, 0.9)
**Delivery assisted by health professional**				
No^†^		--	--	--
Yes		0.7 (0.6, 0.9)	0.8 (0.6, 1.0)	0.7 (0.6, 0.9)
**Child's birth order**				
1^†^		--	--	--
2		0.8 (0.7, 1.0)	0.9 (0.7, 1.0)	0.9 (0.7, 1.1)
3		0.9 (0.8, 1.1)	1.0 (0.8, 1.2)	1.0 (0.8, 1.2)
4+		1.2 (1.0, 1.4)	1.4 (1.1, 1.7)	1.3 (1.0, 1.6)
**Breastfeeding status (month)**				
Never^†^		--	--	--
0–11		0.6 (0.2, 2.0)	0.8 (0.3, 2.5)	0.8 (0.2, 2.4)
12–17		0.7 (0.2, 2.2)	0.8 (0.3, 2.4)	0.7 (0.2, 2.3)
18–23		1.0 (0.3, 3.1)	1.2 (0.4, 3.5)	1.1 (0.3, 3.3)
≥ 24		0.8 (0.3, 2.4)	0.9 (0.3, 2.7)	0.8 (0.3, 2.6)
**Mother's age at childbirth (year)**				
15–24^†^			--	--
25–34			0.7 (0.6, 0.9)	0.8 (0.6, 0.9)
35–49			0.9 (0.6, 1.2)	0.9 (0.7, 1.3)
**Mother's BMI (kg/m^2^)**				
< 18.5			1.4 (1.2, 1.6)	1.3 (1.2, 1.5)
18.5–24.9^†^			--	--
≥ 25.0			0.6 (0.4, 0.8)	0.5 (0.4, 0.8)
**Mother's education**				
No education^†^			--	--
Primary or less			1.0 (0.9, 1.2)	1.0 (0.9, 1.2)
Secondary or higher			0.8 (0.7, 1.0)	0.9 (0.7, 1.1)
Safe drinking water*				
No^†^				--
Yes				1.2 (0.9, 1.5)
**Arsenic in drinking water (parts per billion)**				
≤ 50^†^				--
> 50				1.0 (0.8, 1.3)
**Hygienic toilet**				
No^†^				--
Yes				0.9 (0.8, 1.1)
**Cooking fuel**				
Not highly polluted^†^				--
Highly polluted				1.0 (0.8, 1.4)
**Urban/rural**				
Urban^†^				--
Rural				0.8 (0.7, 0.9)
**Geographic division**				
Barisal^†^				--
Chittagong				0.9 (0.7, 1.2)
Dhaka				0.8 (0.6, 1.0)
Khulna				0.6 (0.4, 0.7)
Rajshahi				0.6 (0.5, 0.8)
Sylhet				0.9 (0.7, 1.2)
**Number of children**	5,911	5,911	5,884	5,363

^†^Reference group

For variable definitions, see Table 1.

### Effects of other risk factors and confounders

Among the controlled variables, a child's age and multiple-birth status have the strongest effects on the risk of a child suffering from growth-stunting. Additionally, this effect is independent of the household wealth status and other maternal and household characteristics (Table 2). When we control for household wealth status and other factors such as antenatal care, delivery type, mother's age at childbirth, mother's BMI, and residence, we find all have statistically significant effects, but these effects are generally small. The adjusted prevalence of adverse growth-stunting is significantly lower in the Khulna and Rajshahi divisions than in any other division (Table 2). We also carried out the above multivariate analysis separately for boys and girls, for urban and rural areas, and for children whose mothers had no education, a primary education or less, and a secondary education or higher. We found that household wealth status has a strong negative effect on childhood adverse growth-stunting in each case (results not shown).

## Discussion

The effects of poverty on a child's nutritional status is a manifestation of physical developmental patterns of children who live in poorer conditions with insufficient food intake, have a higher risk to infection, and who lack access to basic health care [37]. Results of this study illustrate that chronic childhood under-nutrition is a critical problem in Bangladesh, and that children in less wealthy households are at a much greater risk of being undernourished than children in wealthier households. Children in the poorest 20% of households are at more than twice the risk of suffering from adverse childhood growth-stunting than children in the wealthiest 20% of households. This is independent of a child's birth status, age, mother's education and nutritional status, household access to clean water and sanitation, and other important factors. The results hold separately by the gender of a child and by the urban/rural residence of a child. These findings are consistent with the results from previous research in other developing countries [27,28], and provide further evidence that wealth inequality is an important risk factor for chronic childhood under-nutrition.

The lack of a gender differential in adverse growth-stunting in our study indicates that there is no intra-household gender bias in feeding and health care for children in Bangladesh. An increasing pattern in many developing countries of growth-stunting by age is consistent with the typical pattern of increasing prevalence of childhood diseases by age such as diarrhea and acute respiratory infections. [40]. This may partly be due to the beginning of feeding solid foods to a child around 6 months of age, which increases the likelihood of consuming contaminated foods and removes the inherent protection provided by breast milk. Additionally, children begin crawling around this age and are more likely to be carried outdoors, which exposes them to additional infections. Consistent with past research, children of multiple- birth status are more likely to be undernourished than children who are single-births [21]. The association between adverse growth-stunting and higher-order births may be due to competition for food within a household that is likely to be greater in households with more children. In addition, there is a higher proportion of adverse growth-stunting among children who were breastfed for more than one year partly due to the fact that poorer mothers are more likely to continue breastfeeding as a substitute for supplemental feeding. Contrary to the expectation, our analysis finds no significant effects of breastfeeding duration and household water and sanitation conditions on childhood adverse growth-stunting.

In previous research, it has been suggested that a mother's education is one of the more important factors in promoting a family's health and nutrition, increasing household income [39,40]. However, in our analysis, maternal education is found to have little to no effect on adverse childhood growth-stunting; even when we control for a mother's education, this does not significantly alter the effect of household wealth status on growth-stunting. This may be partly due to the majority (69%) of mothers in Bangladesh having a primary education, less than primary education, or no formal education at all.

One potential limitation of this analysis is that it does not control for diet and other health care indicators. However, household wealth status functions mainly through better access to food and health care in affecting childhood nutritional status, for example more wealthy households can afford better food in terms of quality. In the case of adults, the association between nutritional status and household wealth status could be bi-directional and have a reverse-causal relationship. In fact, household wealth status can affect access to food and health care, but undernourished adults whose ability to work is limited will in turn affect the household economic status of the household. In this case, our inability to control for food intake and access to health care is not a major limitation.

Another potential limitation is the cross-sectional design of our analysis. However, due to the fact that the relationship operates basically from household wealth status to childhood growth-stunting, the effects estimated in this study are a good measure of the causal relationship between household wealth status and childhood chronic under-nutrition. Moreover, the study can be criticized for using an indirect measure of household wealth. However, due to the fact that in developing countries like Bangladesh it is hard to obtain reliable income and expenditure data, an asset-based index is generally considered a good proxy for household wealth status. Notwithstanding these limitations, there is evidence of a relationship between household wealth status and others factors and childhood growth-stunting which suggests that improving the health and nutritional status of children in Bangladesh can be realized through expanding and integrating community health and nutritional programs and initiatives targeting the poor. These programs include but are not limited to the Bangladesh Integrated Nutritional Program (BINP) and Program for Bangladesh Poverty Reduction (PBPR).

## Competing interests

The author(s) declare that they have no competing interests.

## Authors' contributions

RH carried out the study design, data management and analysis, and drafted and revised the manuscript. JEB participated in the designing of the study, and in drafting and revising the manuscript. JAB participated in the designing of the study, in managing of the data, and in drafting and revising the manuscript. All authors read and approved the final manuscript.
